# Prognostic role of systemic inflammation response index (SIRI) in patients with pancreatic cancer: a meta-analysis

**DOI:** 10.3389/fonc.2024.1465279

**Published:** 2024-12-11

**Authors:** Huifen Shen, Fei Zuo

**Affiliations:** ^1^ Department of Neurology, Huzhou Central Hospital, Affiliated Central Hospital of Huzhou University, Huzhou, Zhejiang, China; ^2^ Department of Gastroenterology, Huzhou Central Hospital, Affiliated Central Hospital of Huzhou University, Huzhou, Zhejiang, China

**Keywords:** SIRI, pancreatic cancer, prognosis, evidence-based medicine, biomarker

## Abstract

**Background:**

The significance of the systemic inflammation response index (SIRI) in predicting the prognosis of patients with pancreatic cancer (PC) has been extensively explored; however, findings remain controversial. As such, this meta-analysis was performed to more precisely determine the utility of the SIRI in predicting PC prognosis.

**Methods:**

A comprehensive literature search of the PubMed, Web of Science, Embase, and Cochrane Library databases for relevant studies, published up to June 25, 2024, was performed. The primary and secondary endpoints were overall survival (OS) and progression-free survival (PFS), respectively. The prognostic utility of the SIRI in predicting PC prognosis was estimated by calculating pooled hazard ratios (HRs) and corresponding 95% confidence intervals (CIs).

**Results:**

Seven studies comprising 1160 patients were included in the present meta-analysis. Pooled findings revealed that elevated SIRI was as a prominent prognostic marker of OS (HR 2.40 [95% CI 1.88–3.05]; p<0.001) and PFS (HR 1.95 [95% CI 1.19–3.21]; p=0.008) in patients diagnosed with PC. According to subgroup analysis, the SIRI remained an outstanding prognostic marker for OS, irrespective of region, sample size, study center, study design, TNM stage, cancer type, cut-off value, treatment, or survival analysis type (all p<0.05). Moreover, based on subgroup analysis, the SIRI demonstrated significant utility in predicting PFS, irrespective of region and threshold value (p<0.05).

**Conclusion:**

Results of the present meta-analysis revealed that an increased SIRI significantly predicted OS and PFS in patients diagnosed with PC. Considering its cost-effectiveness and availability, the SIRI may be a promising biomarker for predicting prognosis in patients with PC.

## Introduction

Pancreatic cancer (PC) ranks among the most common cancers of the digestive system and is characterized by poor prognosis and limited oncological treatment options (1). The global burden of PC has more than doubled in the past 25 years, ranking it as the seventh major cause of cancer-associated mortality globally (2). According to statistics from GLOBOCAN, 495,773 new cases of PC were diagnosed, with 466,003 related deaths reported worldwide in 2020 (2). Once detected, PC is usually in an advanced stage and cannot be surgically resected in approximately 80% of cases (3). There is only a 20% surgical resection rate in cases of PC that develop local or distant metastases, and metastasis and recurrence often occur even after surgical treatment (4). PC is highly malignant, difficult to diagnose early, and difficult to treat once it has already progressed (5). Pancreatic ductal adenocarcinoma (PDAC) is the most prevalent of PC subtypes, and is the deadliest malignancy, with a five-year survival rate < 8% (6). Consequently, the identification of novel and effective markers for individuals diagnosed with PC is urgently needed.

In recent years, inflammatory and immune responses have been suggested to play crucial roles in cancer progression and development (7). Many hematological parameters have been identified as significant prognostic markers for various cancers, such as lymphocyte-to-monocyte ratio (8), platelet-to-lymphocyte ratio (9), C-reactive protein-to-albumin ratio (10), controlling nutritional status score (CONUT) (11), and fibrinogen-to-albumin ratio (12). The systemic inflammation response index (SIRI) is calculated using neutrophil, lymphocyte, and monocyte counts (13). First proposed in 2016, the SIRI is calculated as neutrophil count × monocyte count/lymphocyte count (13). Recently, the SIRI was demonstrated to be highly significant in predicting the prognosis of various solid tumors, including non-small cell lung (14), breast (15), gastric (16), rectal (17), and hepatoblastoma (18) cancers. The SIRI has been widely analyzed for its prognostic significance in patients diagnosed with PC, although findings remain inconsistent (13, 19–24). As such, we performed a comprehensive literature review and meta-analysis to more precisely define the prognostic utility of the SIRI in patients diagnosed with PC.

## Materials and methods

### Study guideline

The current literature review and meta-analysis was performed in accordance with the Preferred Reporting Items for Systematic Reviews and Meta-Analyses (i.e., “PRISMA”) guidelines (25).

### Search strategy

A comprehensive search of the PubMed, Web of Science, Embase, and Cochrane Library databases for potentially eligible studies, published up to June 25, 2024, was performed using the following search terms: “systemic inflammation response index” or “systemic inflammatory response index” or “SIRI” and “pancreatic cancer” or “pancreatic carcinoma” or “pancreatic tumor” or “pancreatic adenocarcinoma” or “pancreatic neoplasm”. Eligible studies were restricted to those published in English. Additionally, the reference lists of the retrieved studies were manually searched for other potentially eligible studies that fulfilled the inclusion criteria.

### Inclusion and exclusion criteria

Studies fulfilling the following criteria were included: PC diagnosed by histological or cytological examination; reported the relationship between the SIRI and any survival of PC cases; relevant data including hazard ratio (HR) and 95% confidence interval (CI); identification of the threshold SIRI; and available full-text published in English. This meta-analysis utilized the pretreatment measured SIRI, excluding SIRI values assessed at various timepoints such as post-operative or pre/post neoadjuvant chemotherapy.

Case reports, meeting abstracts, letters, comments, and reviews, and studies with duplicate patients and animal studies were excluded.

### Data collection and quality evaluation

Two researchers (HS and FZ) extracted data from the included studies, and disputes were resolved through consensus discussion. The following information was obtained from each included study: first author; publication year; country; sample size; age; sex; study duration; study center; study design; tumor stage; cancer type; threshold SIRI; threshold determination approach; survival outcomes; survival analysis types; survival endpoints; follow-up; and HRs with corresponding 95% CIs. Overall survival (OS) and progression-free survival (PFS) were the primary and secondary endpoints, respectively. Quality assessment was performed using the Newcastle–Ottawa Scale (NOS) (26). The NOS assesses study quality from 3 perspectives: selection, outcome, and comparability. The total NOS score ranges from 0 to 9, with scores ≥ 6 indicating high quality.

### Statistical analysis

The utility/significance of the SIRI for predicting PC prognosis was estimated by calculating combined HRs and corresponding 95% CIs. Heterogeneity among the studies was evaluated using Cochran’s test and the Higgins I^2^ statistic. Results with p ≥ 0.10 and I^2^ ≤ 50% represented no obvious heterogeneity and a fixed-effects model was used to analyze data; otherwise, a random-effects model was adopted. Subgroup analyses according to different factors were performed to detect the sources of heterogeneity for further investigation. Stability of the results was evaluated using sensitivity analysis, in which each study was excluded one-at-a-time (i.e., “leave-one-out” method). Funnel plots were constructed and Begg’s and Egger’s tests were used to evaluate publication bias. Statistical analysis was performed using Stata Release 12.0 (StataCorp LLC, College Station, TX, USA). Differences with p < 0.05 were considered to be statistically significant.

## Results

### Search results

**Figure 1:**
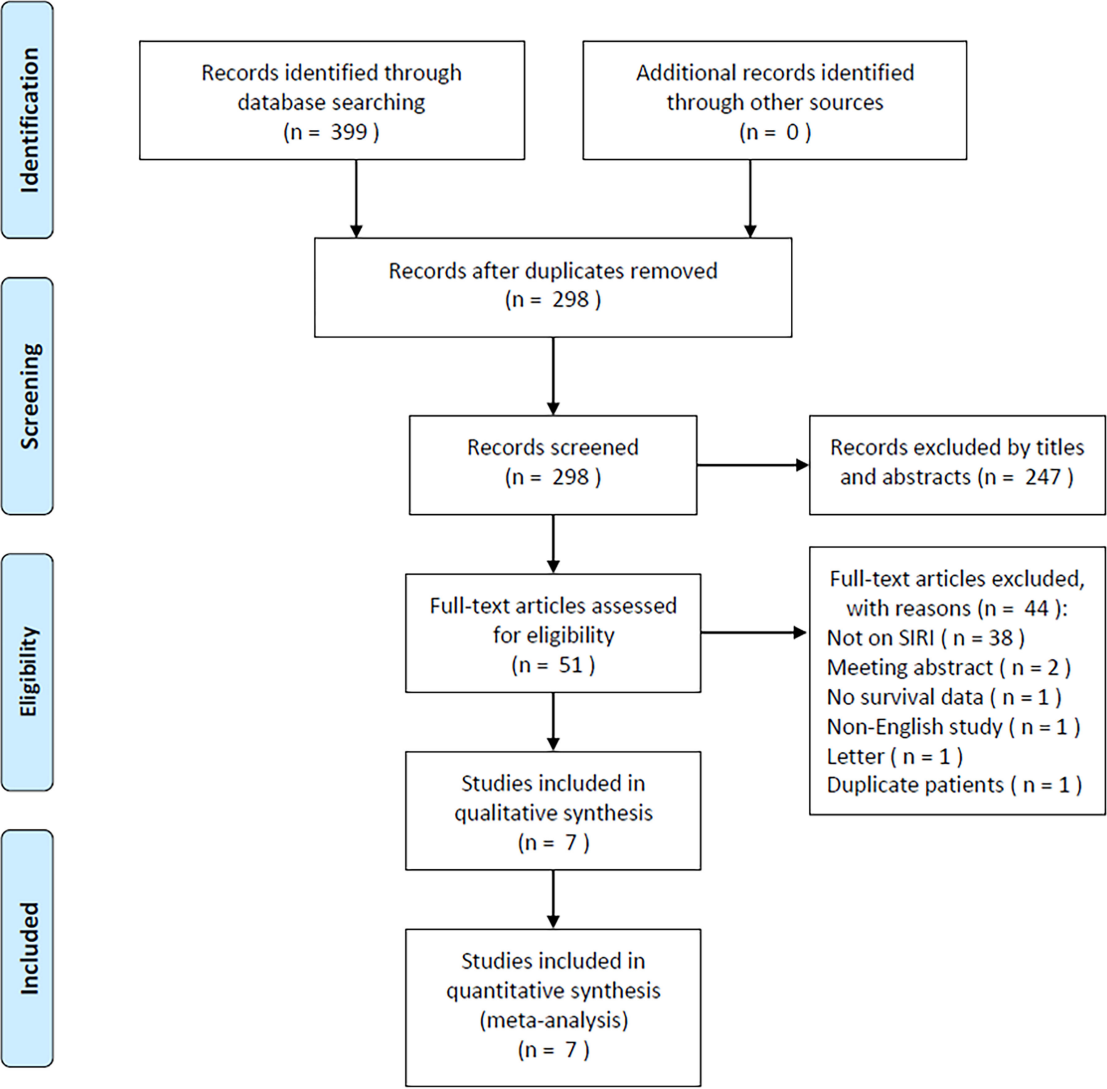
PRISMA flowchart explaining the article selection.

The initial literature search retrieved 399 studies, of which 298 were retained after duplicates were removed (**Figure 1**). After title and abstract review, 247 studies were excluded due to irrelevance and animal studies, and the full texts of 51 studies were further examined. Forty-four studies were excluded for the following reasons: irrelevance to the SIRI (n=38); meeting abstracts (n=2); lack of survival data (n=1); non-English publication (n=1); letters (n=1); and studies with duplicate patients (n=1). Ultimately, 7 studies comprising 1160 patients (13, 19–24) were included in the present meta-analysis (**Figure 1**; **Table 1**).

**Table 1:** Basic characteristics of included studies in this meta-analysis. .

Author	Year	Country	Sample size	Gender (M/F)	Age (years) Median(range)	Study period	Study center	Stude design	TNM stage	Treatment	Cancer type	SIRI cut-off value	Cut-off determination	Survival endpoints	Survival analysis	Follow-up (months) Median(range)	NOS score
Qi, Q.	2016	China	177	108/69	58.8	2009-2010	Single center	Retrospective	III-IV	Chemotherapy	PC	1.8	ROC curve	OS, PFS	Multivariate	1-10	8
Li, S.	2019	China	371	224/147	62(35-84)	2011-2013	Single center	Retrospective	I-III	Surgery	PDAC	0.69	ROC curve	OS, PFS	Multivariate	1-80	7
Topkan, E.	2021	Turkey	152	119/33	52(27-79)	2007-2019	Single center	Retrospective	III-IV	CRT	PDAC	1.8	ROC curve	OS, PFS	Multivariate	18.5(3.2-91.3)	8
Dâmaso, S.	2022	Portugal	112	53/59	71(34-88)	2016-2021	Single center	Retrospective	III-IV	Chemotherapy	PDAC	1.34	ROC curve	OS, PFS	Univariate	8.7(1-52)	7
Kamposioras, K.	2022	UK	138	87/51	62(29-77)	2010-2019	Multicenter	Retrospective	III-IV	Chemotherapy	PDAC	2.35	ROC curve	OS	Multivariate	47.2(0.3-64.9)	8
Kim, J. S.	2022	South Korea	160	92/68	61.8	2006-2019	Single center	Retrospective	I-III	NACT+ surgery	PDAC	0.95	ROC curve	OS, PFS	Univariate	30(1-140)	8
Pacheco-Barcia, V.	2024	Spain	50	32/18	66(32-85)	2020-2023	Multicenter	Prospective	IV	Chemotherapy	PC	2.3	ROC curve	OS, PFS	Univariate	1-48	9

M, male; F, female; SIRI, systemic inflammation response index; OS, overall survival; PFS, progression-free survival; ROC, receiver operating characteristic; CRT, chemoradiotherapy; PDAC, pancreatic ductal adenocarcinoma; NACT, neoadjuvant chemotherapy; TNM, tumor-node-metastasis; NOS, Newcastle-Ottawa Scale.

### Study features

All enrolled studies were published between 2016 and 2024 (**Table 1**). Two were performed in China (13, 19) and 1 each in Turkey (20), Portugal (21), the United Kingdom (22), South Korea (23), and Spain (24). Sample sizes ranged from 50 to 371 (median, 152). There were 5 single-center studies (13, 19–21, 23) and 2 were multicenter trials (22, 24). Six studies had a retrospective design (13, 19–23) and 1 was a prospective trial (24). Four studies recruited patients with TNM stages III-IV (13, 20–22), 2 enrolled patients with stages I-III (19, 23), and 1 included patients with stage IV disease (24). Four studies treated patients with PC with chemotherapy (13, 21, 22, 24), and 1 each used surgery (19), chemoradiotherapy (CRT) (20), and neoadjuvant chemotherapy (NACT) + surgery (23). Five studies included patients with PDAC (19–23), and 2 included patients with PC (13, 24). The threshold SIRI was 0.69–2.35. All studies used receiver operating characteristic (ROC) curve analysis to determine threshold values. All 7 included studies reported the relationship between the SIRI and OS (13, 19–24), whereas 6 presented the significance of the SIRI in predicting PFS (13, 19–21, 23, 24) in PC. Three studies obtained HRs and 95% CIs using univariate regression (21, 23, 24), while 4 used multivariate regression (13, 19, 20, 22). NOS scores ranged from 7 to 9, suggesting high quality (**Table 1**).

### SIRI and OS in PC

**Figure 2:**
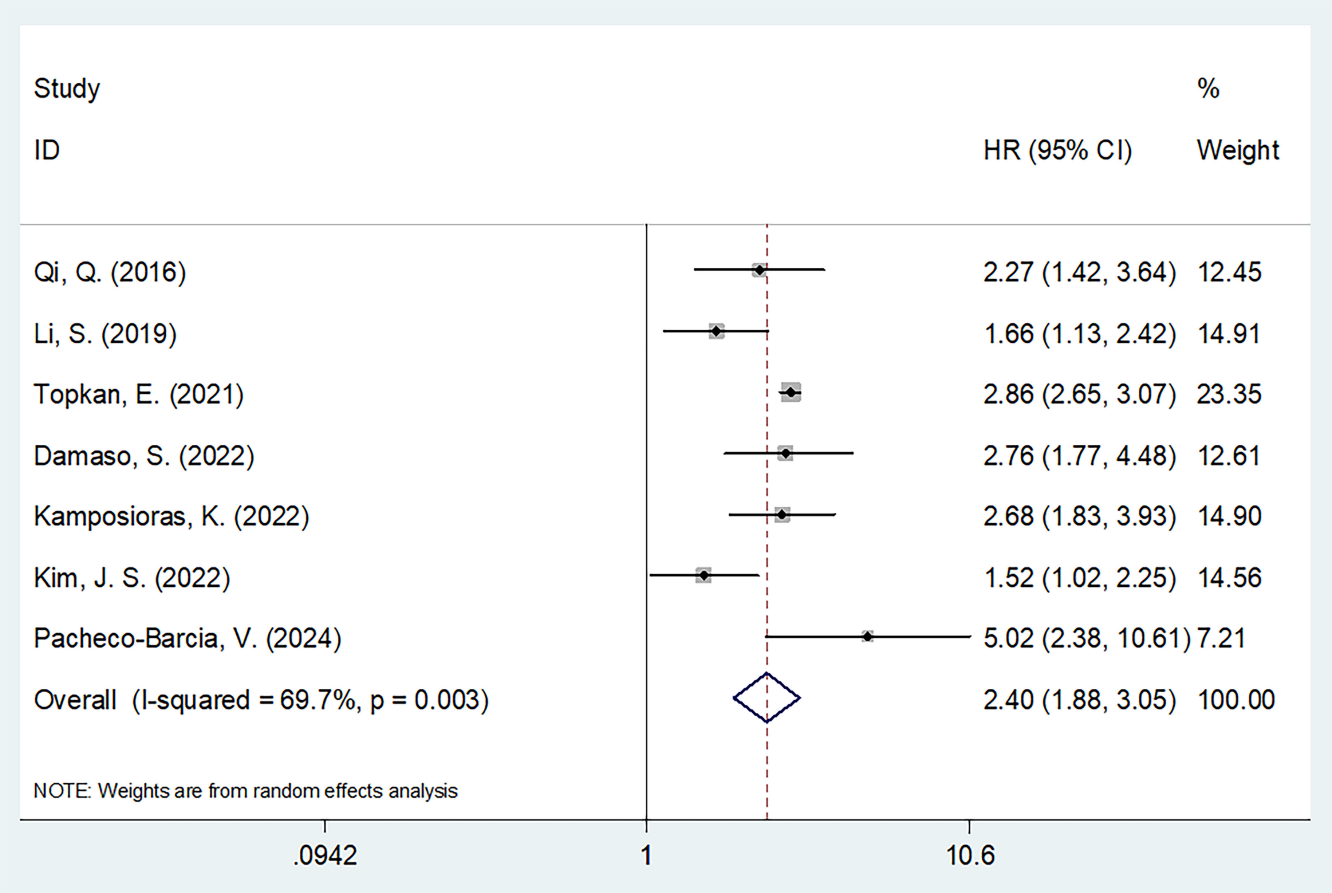
Forest plots of the association between SIRI and overall survival in patients with PC.

Seven studies involving 1160 patients (13, 19–24) reported data regarding the relationship between the SIRI and OS in PC. Due to obvious heterogeneity (I^2^ = 69.7%, p = 0.003), a random-effects model yielded an HR of 2.40 (95% CI 1.88–3.05; p < 0.001), suggesting that an elevated SIRI was the significant prognostic marker for OS in patients with PC (**Figure 2**; **Table 2**). As demonstrated by subgroup analysis, the SIRI remained a significant predictor of OS regardless of region, sample size, study center, study design, TNM stage, cancer type, cut-off value, treatment, or survival analysis type (all p < 0.05) (**Table 2**).

**Table 2:** Subgroup analysis of prognostic value of SIRI for OS in patients with pancreatic cancer.

Subgroups	No. of studies	No. of patients	Effects model	HR (95%CI)	p	Heterogeneity	
						I^2^(%)	Ph
Total	7	1160	Random	2.40(1.88-3.05)	<0.001	69.7	0.003
Region							
Asian	3	708	Fixed	1.74(1.37-2.70)	<0.001	0	0.414
Non-Asian	4	452	Fixed	2.87(2.67-3.08)	<0.001	0	0.512
Sample size							
<150	3	300	Fixed	2.94(2.24-3.88)	<0.001	11.6	0.323
≥150	4	860	Random	2.06(1.42-2.98)	<0.001	82.6	0.001
Study center							
Single center	5	972	Random	2.18(1.61-2.94)	<0.001	78.6	0.002
Multicenter	2	188	Random	3.37(1.86-6.08)	<0.001	53.3	0.143
Study design							
Retrospective	6	1110	Random	2.27(1.77-2.90)	<0.001	71.1	0.004
Prospective	1	50	–	5.02(2.37-10.61)	<0.001	–	–
TNM stage							
I-III	2	531	Fixed	1.59(1.21-2.09)	0.001	0	0.756
III-IV	4	579	Fixed	2.84(2.64-3.04)	<0.001	0	0.803
IV	1	50	–	5.02(2.37-10.61)	<0.001	–	–
Cancer type							
PC	2	227	Random	3.20(1.48-6.89)	0.003	67.5	0.079
PDAC	5	933	Random	2.26(1.70-2.99)	<0.001	76.0	0.002
SIRI cut-off value							
<1.8	3	643	Random	1.86(1.33-2.62)	<0.001	51.3	0.129
≥1.8	4	517	Fixed	2.85(2.66-3.06)	<0.001	5.9	0.364
Treatment							
Chemotherapy	4	477	Fixed	2.76(2.17-3.50)	<0.001	4.0	0.373
Surgery/ NACT+ surgery	2	531	Fixed	1.59(1.21-2.09)	0.001	0	0.756
CRT	1	152	–	2.86(2.66-3.08)	<0.001	–	–
Survival analysis							
Univariate	3	322	Random	2.59(1.38-4.87)	0.003	77.7	0.011
Multivariate	4	838	Random	2.41(1.87-3.11)	<0.001	64.2	0.039

SIRI, systemic inflammation response index; CRT, chemoradiotherapy; PDAC, pancreatic ductal adenocarcinoma; PC, pancreatic cancer; NACT, neoadjuvant chemotherapy; TNM, tumor-node-metastasis.

### SIRI and PFS of PC

**Figure 3:**
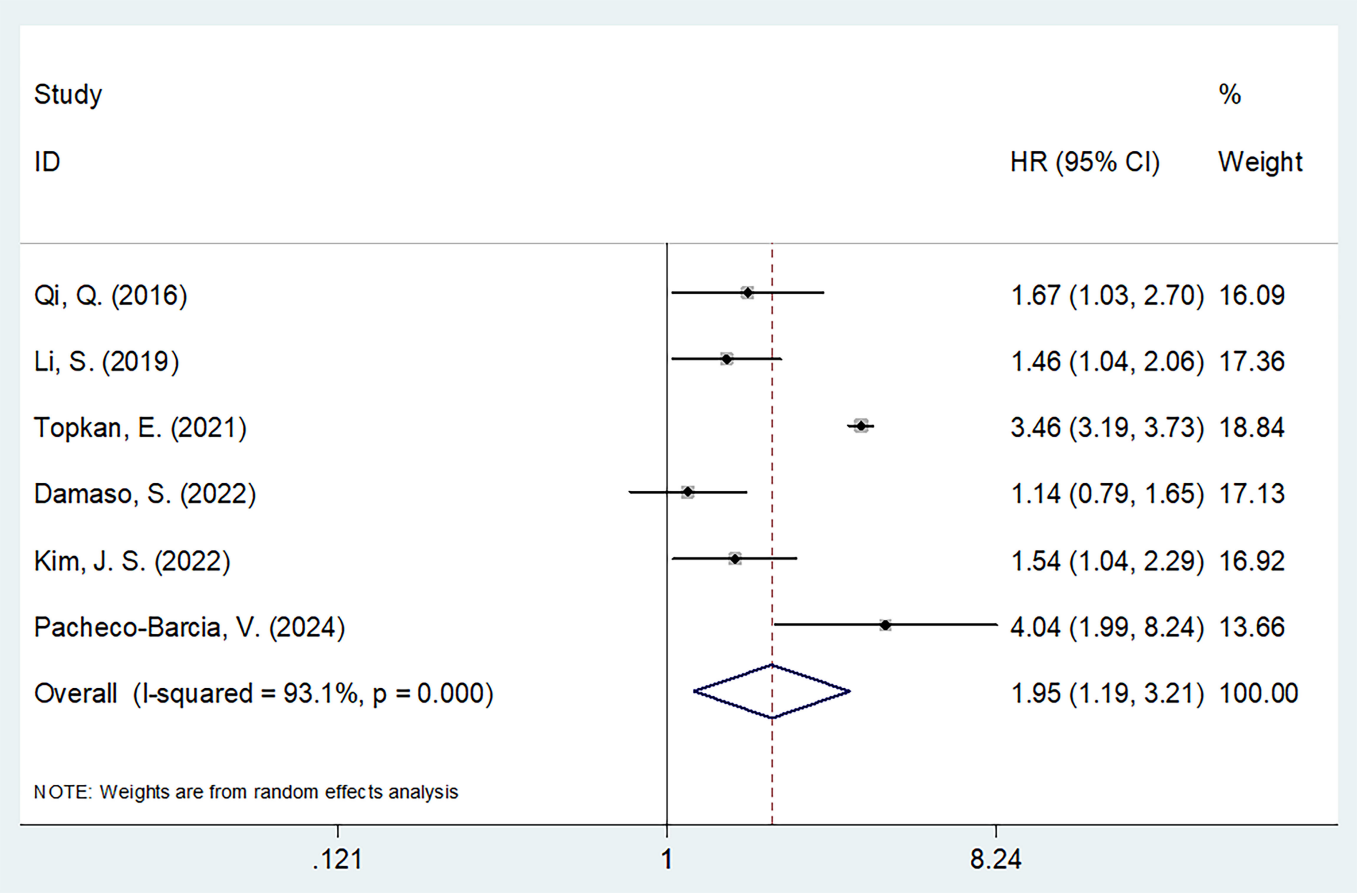
Forest plots of the association between SIRI and progression-free survival in patients with PC.

Six studies comprising 1022 patients (13, 19–21, 23, 24) reported SIRI values for predicting PFS in patients diagnosed with PC. Due to significant heterogeneity (I^2^ = 93.1%, p < 0.001), a random-effects model was used. Based on pooled data, a higher SIRI was markedly associated with dismal PFS in patients with PC (HR 1.95 [95% CI 1.19–3.21]); p = 0.008) (**Figure 3**; **Table 3**). Based on subgroup analysis, the significant prognostic value of the SIRI for PFS remained unaffected by region or cut-off value (p<0.05) (**Table 3**). Additionally, the SIRI still significantly predicted PFS of PC in the following subgroups: sample size ≥ 150 (p = 0.021); multicenter studies (p < 0.001); prospective studies (p < 0.001); TNM stage I-III (p = 0.002) and stage IV (p < 0.001); PDAC histology (p = 0.037); treatment of surgery/NACT+ surgery (p = 0.002) and CRT (p < 0.001); and multivariate survival analysis (p = 0.028) (**Table 3**).

**Table 3:** Subgroup analysis of prognostic value of SIRI for PFS in patients with pancreatic cancer.

Subgroups	No. of studies	No. of patients	Effects model	HR (95%CI)	p	Heterogeneity	
						I^2^(%)	Ph
Total	6	1022	Random	1.95(1.19-3.21)	0.008	93.1	<0.001
Region							
Asian	3	708	Fixed	1.53(1.22-1.93)	<0.001	0	0.905
Non-Asian	3	314	Random	2.47(1.10-5.55)	<0.001	94.0	<0.001
Sample size							
<150	2	162	Random	2.07(0.60-7.12)	0.250	89.5	0.002
≥150	4	860	Random	1.94(1.1-3.40)	0.021	93.2	<0.001
Study center							
Single center	5	972	Random	1.74(1.00-3.03)	0.050	94.5	<0.001
Multicenter	1	50	–	4.04(1.99-8.22)	<0.001	–	–
Study design							
Retrospective	5	972	Random	1.74(1.00-3.03)	0.050	94.5	<0.001
Prospective	1	50	–	4.04(1.99-8.22)	<0.001	–	–
TNM stage							
I-III	2	531	Fixed	1.50(1.15-1.94)	0.002	0	0.846
III-IV	3	441	Random	1.91(0.87-4.20)	0.108	95.1	<0.001
IV	1	50	–	4.04(1.99-8.22)	<0.001	–	–
Cancer type							
PC	2	227	Random	2.50(1.06-5.90)	0.037	75.4	0.044
PDAC	4	795	Random	1.75(0.92-3.33)	0.086	95.5	<0.001
SIRI cut-off value							
<1.8	3	643	Fixed	1.37(1.11-1.69)	0.004	0	0.492
≥1.8	3	379	Random	2.85(1.73-4.69)	<0.001	77.3	0.012
Treatment							
Chemotherapy	3	339	Random	1.86(0.98-3.52)	0.059	79.4	0.008
Surgery/ NACT+ surgery	2	531	Fixed	1.50(1.15-1.94)	0.002	0	0.846
CRT	1	152	–	3.46(3.20-3.74)	<0.001	–	–
Survival analysis							
Univariate	3	322	Random	1.78(0.99-3.22)	0.055	79.1	0.008
Multivariate	3	700	Random	2.08(1.08-4.02)	0.028	93.4	<0.001

SIRI, systemic inflammation response index; CRT, chemoradiotherapy; PDAC, pancreatic ductal adenocarcinoma; PC, pancreatic cancer; NACT, neoadjuvant chemotherapy; TNM, tumor-node-metastasis.

### Sensitivity analysis

**Figure 4:**
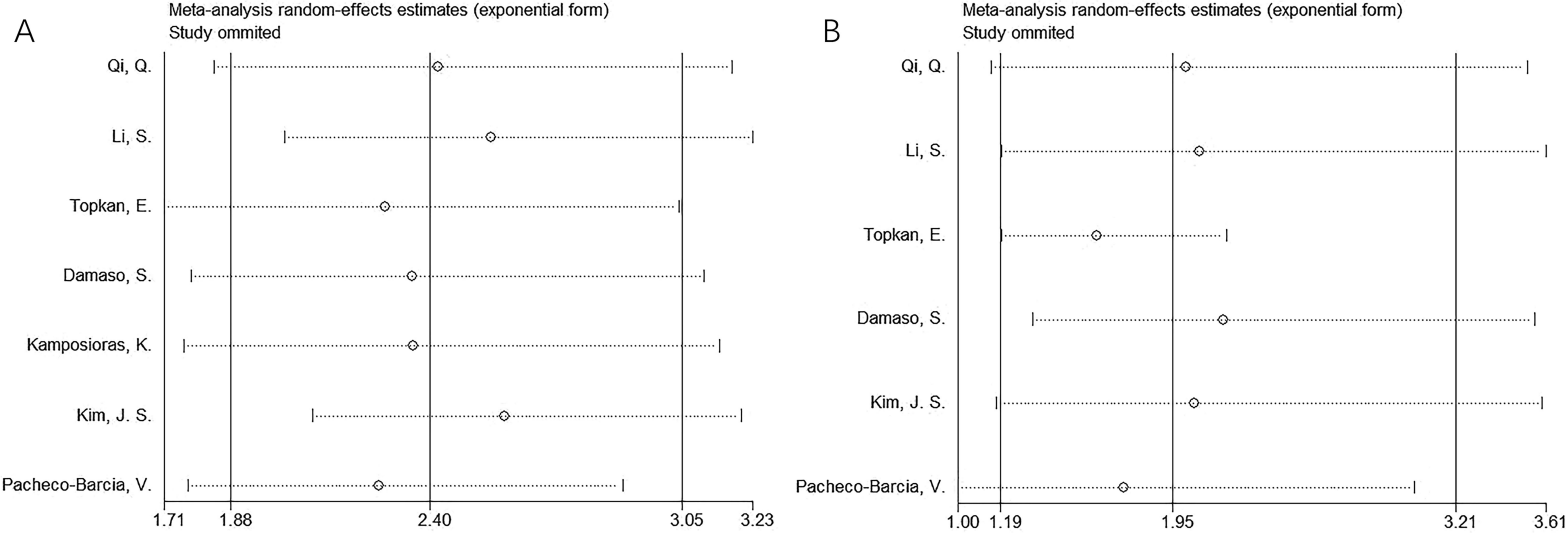
Sensitivity analysis. **(A)** OS; and **(B)** PFS.

Results of sensitivity analyses using the “leave-one-out” method for OS and PFS are reported in **Figure 4**. One study did not demonstrate significant changes in OS or PFS in this meta-analysis, indicating that the findings were reliable (**Figure 4**).

### Publication bias

**Figure 5:**
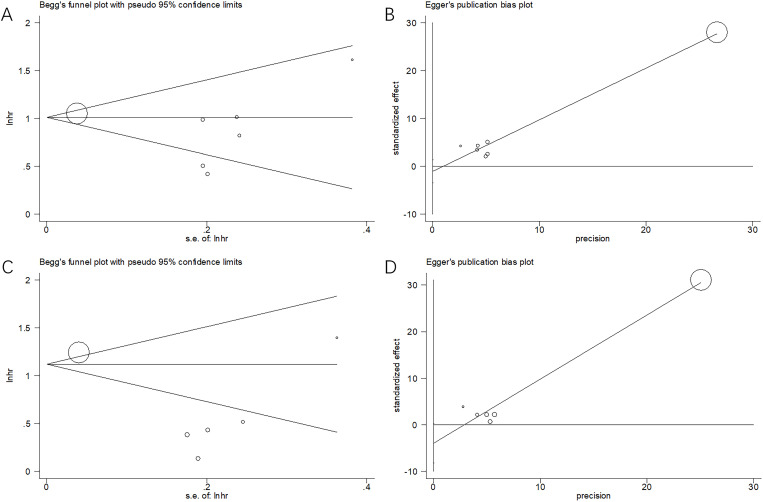
Publication bias test. **(A)** Begg’s test for OS, p=1.000; **(B)** Egger’s test for OS, p=0.305; **(C)** Begg’s test for PFS, p=0.707; and **(D)** Egger’s test for PFS, p=0.060.

Funnel plots and Egger’s and Begg’s tests were used to estimate possible publication bias. Publication bias was not detected for OS (p = 1.000 and p = 0.305 according to Begg’s and Egger’s tests, respectively) and PFS (p = 0.707 and p = 0.060 according to Begg’s and Egger’s tests, respectively) (**Figure 5**).

## Discussion

The efficiency of the SIRI in predicting PC prognosis has been extensively analyzed; however, findings have been inconsistent. For example, a high SIRI has been suggested to be a significant prognostic marker of PC in some studies (13, 19, 20, 22–24). In contrast, some clinicians have failed to determine a relationship between the SIRI and PC prognosis (21). These inconsistencies prevent the clinical application of SIRI for PC prognostication.

In this meta-analysis, we aggregated data from 7 studies involving 1160 patients (13, 19–24) to more clearly define the prognostic utility of the SIRI. Based on our results, a higher SIRI significantly predicted OS and PFS in patients with PC. Moreover, the role of the SIRI in predicting OS and PFS remained unaffected by geographical region and cut-off values in PC. As verified by publication bias and sensitivity analyses, our findings were stable. Collectively, a higher SIRI significantly predicted the short- and long-term prognoses of patients with PC. The SIRI may a candidate biomarker for predicting PC prognosis due to its cost-effectiveness and availability. To the best of our knowledge, this meta-analysis is the first to explore the utility of the SIRI in predicting PC prognosis.

We computed the SIRI using neutrophil, lymphocyte, and monocyte counts. Consequently, a higher SIRI may be due to higher neutrophil/monocyte counts and/or lower lymphocyte counts. Although the precise mechanisms underlying the significance of the SIRI in predicting PC prognosis remain largely unclear, they are interpreted as follows. First, it is known that neutrophils produce growth factors, chemokines and cytokines that can promote angiogenesis, such as transforming growth factor-beta, vascular endothelial growth factor, matrix metalloproteinases, and interleukin (IL)-6, IL-8, and IL-12 (27). In addition to secreting cytokines, neutrophils also produce proteases, including matrix metalloproteinases, cysteine cathepsins, and serine proteases (28, 29). Because these proteases disrupt cell connections and degrade the extracellular matrix and basement membrane proteins, tumor cells can migrate more easily (30). Second, monocytes may affect tumor occurrence by differentiating into tumor-associated macrophages (TAMs). Chemokines and cytokines in the tumor microenvironment exert a chemotactic effect on TAMs, including tumor necrosis factor-α and monocyte chemoattractant protein-1, among others (31). Furthermore, monocytes can inhibit antigen- and mitogen-induced lymphocyte proliferation, impair lymphocyte-dependent antitumor defenses, and suppress antitumor immunity (32). Third, lymphocytes, particularly tumor-infiltrating lymphocytes (TILs), are important for cell-mediated immunity against tumors (33). Lower lymphocyte counts can weaken the systemic immune system; therefore, tumor cells can evade immune surveillance, ultimately enhancing their malignant behavior (34).

Results of the present meta-analysis have important implications for clinical practice. First, variations in the follow-up duration of the included studies may have affected the prognostic role of the SIRI. Therefore, adequate follow-up is needed for the post-treatment management of PC. Second, the SIRI may vary during the treatment process for PC. In this meta-analysis, we adopted pretreatment blood test parameters to calculate the SIRI. Moreover, infections, trauma, and immune-related diseases should be excluded when the SIRI is calculated because they can affect specific immunological indices. Third, this meta-analysis included only the pretreatment SIRI. Changes in SIRI scores before and after treatment may provide prognostic value, which should be explored in future studies. Subgroup analysis indicated that an increased SIRI was significantly associated with poor OS and PFS in patients with PC who underwent surgery or NACT + surgery. However, an elevated SIRI was a significant prognostic marker for poor OS―but not PFS―in patients with PC treated with chemotherapy. Therefore, in patients with resectable PC, the SIRI remains a significant prognostic indicator of both OS and PFS.

Notably, SIRI cut-off values varied among the included studies, ranging from 0.69 to 2.35, with a median value of 1.8; as such, 1.8 was adopted as the cut-off value in the subgroup analysis. The carbohydrate antigen 19-9 (CA 19-9) is a glycoprotein found on the cell surface of the pancreatic ductal cells (35). A wide range of benign diseases, such as cholestasis, and malignant diseases, mainly PDAC, overexpress CA19-9 (36). Preoperative serum levels of CA 19-9 are associated with both occult metastasis detection during surgery and worse disease-free survival (DFS), even in resectable PDAC (37). For patients with PDAC, CA 19-9 is considered to be the main biological parameter to assess its biological resectability (38). Whether the combination of SIRI and CA19-9 could enhance the prognostic efficiency for PDAC patients is needed to be investigated in future studies.

Recently, SIRI is widely suggested with prognostic significance for different cancer types by meta-analysis (39–43). As reported by Zhang et al. (39), a higher SIRI estimated poor OS and PFS in hepatocellular carcinoma cases in a meta-analysis of 10 studies. Ren et al. (41) conducted a meta-analysis of 6 studies and found that a higher SIRI value was consistently related to poor OS and DFS in patients with gastric cancer. In addition, another meta-analysis enrolling 3187 patients reported that the SIRI independently predicted OS in nasopharyngeal carcinoma (42). In a recent meta-analysis involving 2997 cases, a higher SIRI was markedly related to poor OS but not poor DFS in breast cancer (40). Our meta-analysis is consistent with results regarding the prognostic utility of the SIRI in other cancer types.

However, the present study had several limitations, the first of which was the small sample size, with only 7 studies included. Second, most included studies were retrospective in design, which may have introduced selection bias due to the inherent nature of such designs. Third, the threshold SIRI was not uniform among the included studies, which may have contributed to heterogeneity. Fourth, it is important to note that many non-specific biological processes may affect the cell counts necessary to calculate SIRI (pathology, cancer, infection, inflammation, etc.). Given these limitations, large-scale prospective studies should be conducted for further validation.

## Conclusions

In summary, results of the present meta-analysis demonstrated that an elevated SIRI significantly predicted OS and PFS in patients diagnosed with PC. Considering its cost-effectiveness and availability, the SIRI may be a promising prognostic biomarker in this patient population.

## Data Availability

The original contributions presented in the study are included in the article/supplementary material. Further inquiries can be directed to the corresponding author/s.

## References

[1] Mizrahi JD Surana R Valle JW Shroff RT . Pancreatic cancer. Lancet. (2020) 395:2008–20. doi: 10.1016/S0140-6736(20)30974-0 32593337

[2] Sung H Ferlay J Siegel RL Laversanne M Soerjomataram I Jemal A . Global cancer statistics 2020: GLOBOCAN estimates of incidence and mortality worldwide for 36 cancers in 185 countries. CA: Cancer J Clin. (2021) 71:209–49. doi: 10.3322/caac.21660 33538338

[3] Klein AP . Pancreatic cancer epidemiology: understanding the role of lifestyle and inherited risk factors. Nat Rev Gastroenterol Hepatol. (2021) 18:493–502. doi: 10.1038/s41575-021-00457-x 34002083 PMC9265847

[4] Wood LD Canto MI Jaffee EM Simeone DM . Pancreatic cancer: pathogenesis, screening, diagnosis, and treatment. Gastroenterology. (2022) 163:386. doi: 10.1053/j.gastro.2022.03.056 35398344 PMC9516440

[5] Park W Chawla A O’Reilly EM . Pancreatic cancer: A review. Jama-Journal Am Med Assoc. (2021) 326:851–62. doi: 10.1001/jama.2021.13027 PMC9363152 34547082

[6] Halbrook CJ Lyssiotis CA di Magliano MP Maitra A . Pancreatic cancer: Advances and challenges. Cell. (2023) 186:1729–54. doi: 10.1016/j.cell.2023.02.014 PMC10182830 37059070

[7] Coussens LM Werb Z . Inflammation and cancer. Nature. (2002) 420:860–7. doi: 10.1038/nature01322 PMC2803035 12490959

[8] Hutterer GC Stoeckigt C Stojakovic T Jesche J Eberhard K Pummer K . Low preoperative lymphocyte-monocyte ratio (LMR) represents a potentially poor prognostic factor in nonmetastatic clear cell renal cell carcinoma. Urologic Oncol. (2014) 32:1041–8. doi: 10.1016/j.urolonc.2014.04.001 25027686

[9] Fu Y Chen X Song Y Huang X Chen Q Lv X . The platelet to lymphocyte ratio is a potential inflammatory marker predicting the effects of adjuvant chemotherapy in patients with stage II colorectal cancer. BMC Cancer. (2021) 21:792. doi: 10.1186/s12885-021-08521-0 34238262 PMC8268489

[10] Sugimoto A Toyokawa T Miki Y Yoshii M Tamura T Sakurai K . Preoperative C-reactive protein to albumin ratio predicts anastomotic leakage after esophagectomy for thoracic esophageal cancer: a single-center retrospective cohort study. BMC Surg. (2021) 21:348. doi: 10.1186/s12893-021-01344-7 34548054 PMC8454123

[11] Xiao Q Li X Duan B Li X Liu S Xu B . Clinical significance of controlling nutritional status score (CONUT) in evaluating outcome of postoperative patients with gastric cancer. Sci Rep. (2022) 12:93. doi: 10.1038/s41598-021-04128-4 34997105 PMC8742112

[12] Sun H Ma J Lu J Yao ZH Ran HL Zhou H . Fibrinogen-to-albumin ratio predicts overall survival of hepatocellular carcinoma. World J Gastrointest Oncol. (2023) 15:1662–72. doi: 10.4251/wjgo.v15.i9.1662 PMC10514720 37746650

[13] Qi Q Zhuang L Shen Y Geng Y Yu S Chen H . A novel systemic inflammation response index (SIRI) for predicting the survival of patients with pancreatic cancer after chemotherapy. Cancer. (2016) 122:2158–67. doi: 10.1002/cncr.30057 27152949

[14] Wang H Li W . Prognostic significance of SIRI in patients with late-stage lung adenocarcinoma receiving EGFR-TKI treatment. Curr Probl Cancer. (2024) 50:101099. doi: 10.1016/j.currproblcancer.2024.101099 38723294

[15] Wu HY Lin CY Tzeng YD Hung CC Liu SI Yin CH . Preoperative systemic inflammation response index: Clinicopathologic predictor of pathological complete response in HER2-positive breast cancer patients receiving neoadjuvant systemic therapy. J Chin Med Association: JCMA. (2024) 87:226–35. doi: 10.1097/jcma.0000000000001034 PMC12718766 38095571

[16] Ren JY Wang D Zhu LH Liu S Yu M Cai H . Combining systemic inflammatory response index and albumin fibrinogen ratio to predict early serious complications and prognosis after resectable gastric cancer. World J Gastrointest Oncol. (2024) 16:732–49. doi: 10.4251/wjgo.v16.i3.732 PMC10989372 38577468

[17] Ding Y Liu Z Li J Niu W Li C Yu B . Predictive effect of the systemic inflammation response index (SIRI) on the efficacy and prognosis of neoadjuvant chemoradiotherapy in patients with locally advanced rectal cancer. BMC Surg. (2024) 24:89. doi: 10.1186/s12893-024-02384-5 38481180 PMC10935841

[18] Zheng C Ye S Liu W Diao M Li L . Prognostic value of systemic inflammation response index in hepatoblastoma patients receiving preoperative neoadjuvant chemotherapy. Front Oncol. (2023) 13:1276175. doi: 10.3389/fonc.2023.1276175 37901310 PMC10613067

[19] Li S Xu H Wang W Gao H Li H Zhang S . The systemic inflammation response index predicts survival and recurrence in patients with resectable pancreatic ductal adenocarcinoma. Cancer Manag Res. (2019) 11:3327–37. doi: 10.2147/cmar.S197911 PMC6489619 31114368

[20] Topkan E Selek U Pehlivan B Kucuk A Haksoyler V Kilic Durankus N . The prognostic significance of novel pancreas cancer prognostic index in unresectable locally advanced pancreas cancers treated with definitive concurrent chemoradiotherapy. J Inflammation Res. (2021) 14:4433–44. doi: 10.2147/jir.S329611 PMC8427684 34511977

[21] Dâmaso S Paiva R Pinho I Esperança-Martins M Brás RL Alvim CM . Systemic inflammatory response index is a prognostic biomarker in unresectable pancreatic adenocarcinoma and identifies patients for more intensive treatment. Memo-Magazine Eur Med Oncol. (2022) 15:246–52. doi: 10.1007/s12254-022-00829-2

[22] Kamposioras K Papaxoinis G Dawood M Appleyard J Collinson F Lamarca A . Markers of tumor inflammation as prognostic factors for overall survival in patients with advanced pancreatic cancer receiving first-line FOLFIRINOX chemotherapy. Acta Oncol (Stockholm Sweden). (2022) 61:583–90. doi: 10.1080/0284186x.2022.2053198 35392758

[23] Kim JS Choi M Kim SH Hwang HK Lee WJ Kang CM . Systemic inflammation response index correlates with survival and predicts oncological outcome of resected pancreatic cancer following neoadjuvant chemotherapy. Pancreatology. (2022) 22:987–93. doi: 10.1016/j.pan.2022.08.009 36064516

[24] Pacheco-Barcia V Custodio-Cabello S Carrasco-Valero F Palka-Kotlowska M Mariño-Mendez A Carmona-Bayonas A . Systemic Inflammation Response Index and weight loss as prognostic factors in metastatic pancreatic cancer: A concept study from the PANTHEIA-SEOM trial. World J Gastrointest Oncol. (2024) 16:386–97. doi: 10.4251/wjgo.v16.i2.386 PMC10900150 38425396

[25] Moher D Liberati A Tetzlaff J Altman DG Grp P . Preferred reporting items for systematic reviews and meta-analyses: the PRISMA statement. J Clin Epidemiol. (2009) 62:1006–12. doi: 10.1016/j.jclinepi.2009.06.005 19631508

[26] Stang A . Critical evaluation of the Newcastle-Ottawa scale for the assessment of the quality of nonrandomized studies in meta-analyses. Eur J Epidemiol. (2010) 25:603–5. doi: 10.1007/s10654-010-9491-z 20652370

[27] Templeton AJ McNamara MG Šeruga B Vera-Badillo FE Aneja P Ocaña A . Prognostic role of neutrophil-to-lymphocyte ratio in solid tumors: a systematic review and meta-analysis. J Natl Cancer Institute. (2014) 106:dju124. doi: 10.1093/jnci/dju124 24875653

[28] Egeblad M Werb Z . New functions for the matrix metalloproteinases in cancer progression. Nat Rev Cancer. (2002) 2:161–74. doi: 10.1038/nrc745 11990853

[29] Mohamed MM Sloane BF . Cysteine cathepsins: multifunctional enzymes in cancer. Nat Rev Cancer. (2006) 6:764–75. doi: 10.1038/nrc1949 16990854

[30] Joyce JA Pollard JW . Microenvironmental regulation of metastasis. Nat Rev Cancer. (2009) 9:239–52. doi: 10.1038/nrc2618 PMC3251309 19279573

[31] Chanmee T Ontong P Konno K Itano N . Tumor-associated macrophages as major players in the tumor microenvironment. Cancers (Basel). (2014) 6:1670–90. doi: 10.3390/cancers6031670 PMC4190561 25125485

[32] Pollard JW . Tumour-educated macrophages promote tumour progression and metastasis. Nat Rev Cancer. (2004) 4:71–8. doi: 10.1038/nrc1256 14708027

[33] Man YG Stojadinovic A Mason J Avital I Bilchik A Bruecher B . Tumor-infiltrating immune cells promoting tumor invasion and metastasis: existing theories. J Cancer. (2013) 4:84–95. doi: 10.7150/jca.5482 23386907 PMC3564249

[34] Durgeau A Virk Y Corgnac S Mami-Chouaib F . Recent advances in targeting CD8 T-cell immunity for more effective cancer immunotherapy. Front Immunol. (2018) 9:14. doi: 10.3389/fimmu.2018.00014 29403496 PMC5786548

[35] Barton JG Bois JP Sarr MG Wood CM Qin R Thomsen KM . Predictive and prognostic value of CA 19-9 in resected pancreatic adenocarcinoma. J gastrointestinal surgery: Off J Soc Surg Alimentary Tract. (2009) 13:2050–8. doi: 10.1007/s11605-009-0849-z 19756875

[36] Lee T Teng TZJ Shelat VG . Carbohydrate antigen 19-9 - tumor marker: Past, present, and future. World J Gastrointest Surg. (2020) 12:468–90. doi: 10.4240/wjgs.v12.i12.468 PMC7769746 33437400

[37] Bhandare MS Gupta V Chaudhari V Nandy K Ostwal V Ramaswamy A . Differential impact of incrementally elevated CA 19-9 levels on prognosis of resected pancreatic ductal adenocarcinoma. HPB: Off J Int Hepato Pancreato Biliary Assoc. (2024) 26:1237–47. doi: 10.1016/j.hpb.2024.06.004 38944571

[38] Isaji S Mizuno S Windsor JA Bassi C Fernández-Del Castillo C Hackert T . International consensus on definition and criteria of borderline resectable pancreatic ductal adenocarcinoma 2017. Pancreatology. (2018) 18:2–11. doi: 10.1016/j.pan.2017.11.011 29191513

[39] Zhang S Tang Z . Prognostic and clinicopathological significance of systemic inflammation response index in patients with hepatocellular carcinoma: a systematic review and meta-analysis. Front Immunol. (2024) 15:1291840. doi: 10.3389/fimmu.2024.1291840 38469315 PMC10925676

[40] Zhang S Cheng T . Prognostic and clinicopathological value of systemic inflammation response index (SIRI) in patients with breast cancer: a meta-analysis. Ann Med. (2024) 56:2337729. doi: 10.1080/07853890.2024.2337729 38569199 PMC10993763

[41] Ren JY Xu M Niu XD Ma SX Jiao YJ Wang D . Systemic inflammatory response index is a predictor of prognosis in gastric cancer patients: Retrospective cohort and meta-analysis. World J Gastrointest Surg. (2024) 16:382–95. doi: 10.4240/wjgs.v16.i2.382 PMC10921201 38463377

[42] Wang L Qin X Zhang Y Xue S Song X . The prognostic predictive value of systemic immune index and systemic inflammatory response index in nasopharyngeal carcinoma: A systematic review and meta-analysis. Front Oncol. (2023) 13:1006233. doi: 10.3389/fonc.2023.1006233 36816962 PMC9936064

[43] Zhou Q Su S You W Wang T Ren T Zhu L . Systemic inflammation response index as a prognostic marker in cancer patients: A systematic review and meta-analysis of 38 cohorts. Dose-response: Publ Int Hormesis Soc. (2021) 19:15593258211064744. doi: 10.1177/15593258211064744 PMC8689621 34987341

